# Kidney Disease1Read at the fourth annual meeting of the National Association of Pension Examining Surgeons, Chicago, Ill., July 7-8, 1905.

**Published:** 1905-09

**Authors:** Nelson W. Wilson

**Affiliations:** Buffalo, N. Y., 49 Niagara Street


### Kidney Disease.[1]

By NELSON W. WILSON, M. D., Buffalo, N. Y.

A FEATURE of examinations for pensions which stands out prominently in a majority of cases is the universal claim of diseases affecting the urinary tract. There are few orders which do not have some reference to the kidneys, especially in the claims of veterans of the civil war. When one considers the ravages of old age, with its attendant aches and pains and the generally obscure knowledge on the part of the layman who ascribes all backache to disease of the kidneys, this is not surprising. At the same time it is quite likely, more than probable, in fact, that in a great many instances these claims, some of which are really remarkable, are entered by pension attorneys whose zeal in the interests of their clients is only equaled by their woeful lack of even the slightest knowledge of what really constitutes disease of the kidneys and urinary tract, although statements frequently made by claimants undergoing examination reveal a suspiciously superficial knowledge of pathologic conditions in connection with the urinary tract.

It is not the intention of this paper to deal with any deep-laid facts of pathologic import or to present any discovery of revolutionising character; but rather to call attention to the methods of examination and diagnosis in use by the Buffalo Board of Pension Examining Surgeons with a view to securing the fullest measure of justice to the soldier and the most ample protection to the government. Merely as an index to the scope of the examinations I quote some of the claims taken from recent orders: "Kidney disease" alone in all its simple complexity is a frequent claim. Then there is "disease of the kidneys and resulting backache," closely followed by "diseases of the urinary organs, bladder, and kidneys." "Results of kidney disease and resulting disease of the urine" was one claim, rather terrifying on its face, but which proved very harmless when the real cause was discovered. Probably the most remarkable "shot-gun" claim was one which presented for consideration "Bright's disease, disease of the kidneys, and diabetes." Examination of this case failed to show any disease of the kidneys; there was neither albumin nor sugar demonstrated after repeated tests and the claimant admitted that his diseases had been diagnosticated by one of the "specialists" in Buffalo whose portrait heads a lurid advertisement in all the newspapers of that city.

1. Read at the fourth annual meeting of the National Association of Pension Examining Surgeons, Chicago, Ill., July 7-8, 1905.

The frequency of claims for disease of the urinary tract, including the kidneys makes a painstaking examination in this direction necessary in every case, and at Buffalo this is carried out to its fullest extent whether a claim is advanced or not, because of the liability of the aged to renal deficiency and disease.

The system of examination adopted by the Buffalo board is so far as we can make it, complete. The claimant is received and given the superficial examination required by the certificate blank. Next, his history is taken and he is required to give a brief but comprehensive statement of each recorded disability. He is then asked regarding any additional disabilities; organs being specified. From this examination he goes to the detailed physical examination in which he is stripped and all recorded disabilities dealt with in detail. Again he is questioned regarding additionals and even when there is no claim search is made for any serious affection which might have a bearing on his case. From this the claimant goes to a thorough examination of the eyes, ears, nose, and throat as a routine procedure when any affection of these organs is claimed or suspected, and in many cases this special examination is made whether claimed or not, for the purpose of tracing out obscure conditions elsewhere. The last acts of the physical examination are a careful search for evidences of venereal disease and the securing of a specimen of urine for analysis which is completed before the next case is begun.

The Buffalo board employs a stenographer and at the conclusion of an examination each member dictates his findings, a consultation is held on ratings and each case is completed before the claimant is allowed to depart. This routine may appear slow, but we consider accuracy and justice to the government and the soldier paramount.

The examination of the urine is merely one of the features of diagnosicating alleged urinary tract disease. In every case before the claimant is allowed to dress a digital examination is made of the prostate and where there is the slightest evidence to warrant it, of the seminal vesicles. All enlargements of the prostate are noted in percentages and the enlargement is specifically located. If it encroaches on the vesical space that fact is mentioned as having a bearing directly on an alkaline urine with burning and urgent frequency. Urine for examination is collected from each individual case in a clean tumbler. If the claimant at the time of examination is unable to furnish a specimen he is required to wait until he can and there is no deviation from the rule that each sample must be voided in the presence of a member of the board. There is a two-fold advantage in requiring the claimant to wait until he can furnish a sample of urine. In the first place a report is incomplete if an analysis of urine is lacking; and the waiting either confirms or disproves a not infrequent statement that urination is compulsory "every few minutes."

The sample is measured; its specific gravity and reaction are taken; its color is recorded and where there is a deviation from the normal transparency that fact is noted as being more important than a mere change in the color. Sugar is examined for by the Fehling test in every case. In a sample with a specific gravity below 1025 a negative first test is considered sufficient, but search is always made for the cause of the increase is specific gravity. Above 1025 every sample is tested and retested each time with a clean set of test tubes until sugar is found or until it is satisfactorily established that it is not present. For albumin mere traces of which are frequently found the nitric acid float test is the routine. If albumin is not developed—no matter what the specific gravity of the sample, a further test is always made with ferrocyanid of potash. This is a test of great delicacy indicating a most minute quantity of albumin, and shows nothing but albumin. It might be used as a qualitative test in itself, but we use it as a confirmatory test because of the ease with which the relative percentage of albumin can be determined with nitric acid. If the albumin ring is 1/8 to 1-12 of an inch broad and it is not granular there is, relatively, 1/8 of 1 per cent. of albumin; if the ring is 1/4 to 1/6 of an inch broad the percentage is 1/2 of 1 per cent. If the band is granular and flocculent and sinks, and if when stirred the mass looks like sour cream the percentage of albumin is from 1 per cent. to 2 per cent. In every case where albumin is found search is made for its probable cause, with particular reference to the heart. If there is no heart affection a portion of the sample is put into a sterile bottle and later in the day the sample is centrifuged at the office and a microscopic search made for casts and other evidences of renal involvement. The results of this examination are sent to the stenographer to be inserted in the report. This may not be required by the bureau, but it is done because of the frequency of claims for urinary trouble, the effort of the board being to determine beyond peradventure the presence or absence of true kidney lesion.

Much can be learned from the odor of urine to which especial attention is paid. The ammoniac urine, with an enlarged prostate tells its own story in spite of reiteration of the claim for kidney disease. The total solids are readily determined by simple multiplication and are a good index of elimination. When there is a liver claim, as is so frequently the case now in Spanish war cases, bile pigments are sought and especial attention is paid to color, reaction, and specific gravity. Blood and pus are easily recognised when they appear in pathologic quantities. Urea is determined relatively with a watch glass and a drop of nitric acid. The method is this: On a clean watch glass place a drop of urine. To this add a drop of nitric acid so the two drops just touch. If crystals form at the edge of the mixture in from 20 to 30 minutes the amount of urea is normal; if in less than 20 minutes there is an excess; if more than 30 minutes there is a deficiency. In nerve and intestinal claims indoxyl is readily determined by the hydrochloric acid test. In rheumatism particular attention is paid to color, acidity and total solids.

In over 500 cases in which kidney disease was claimed only 1 per cent had positive renal disease demonstrated by microscope and urine analysis. Over 50 per cent. came to the examination with unshakable belief that they suffered from some severe form of disease of the urinary tract. In old men in every case of kidney claim hypertrophy of the prostate was found, giving rise to the urgency and frequency of urination, the usual basis of claim for kidney disease. In no case where pain in the back was the chief symptom was there any urinary indications of kidney or bladder disease; the pain in the back in each case being due to lumbago, as demonstrated by pain on pressure and muscular atrophy.

The value and the importance of making a careful analysis of urine as a routine procedure has lately been demonstrated in three cases where no claim was made for kidney disease or any disturbance of the urinary tract. In one case, sugar in large quantity was found and in the other two, albumin; in each case additionals were added to the certificate as disabilities of a pensionable character. These cases, as a matter of justice to the old soldier, showed the wisdom of rigid urinary examination.

Summarised, the majority of claims for kidney disease are traceable to physiologic hypertrophy of the prostate and lumbago, the former giving the urinary symptoms and the latter the "pain in the kidneys" so tenaciously complained of.

We believe the best interests of the service demand the exercise of common sense and justice in considering the disabilities of the old soldier, and not mere bald scientific research. Our constant effort is to give the Washington office the fullest information in connection with each case, good, bad, and indifferent, in order that the work of review may be facilitated, bearing in mind that there is due the government protection and to the soldier,—justice.

49 Niagara Street.

